# Relationship of Excess Weight with Clinical Activity and Dietary Intake Deficiencies in Systemic Lupus Erythematosus Patients

**DOI:** 10.3390/nu11112683

**Published:** 2019-11-06

**Authors:** Mónica R. Meza-Meza, Barbara Vizmanos-Lamotte, José Francisco Muñoz-Valle, Isela Parra-Rojas, Marta Garaulet, Bertha Campos-López, Margarita Montoya-Buelna, Sergio Cerpa-Cruz, Erika Martínez-López, Edith Oregon-Romero, Ulises De la Cruz-Mosso

**Affiliations:** 1 Instituto de Investigación en Ciencias Biomédicas, Centro Universitario de Ciencias de la Salud, Universidad de Guadalajara, Guadalajara, Jalisco 44340, Mexico; 2 Programa de Doctorado en Ciencias Biomédicas, Centro Universitario de Ciencias de la Salud, Universidad de Guadalajara, Guadalajara, Jalisco 44340, Mexico; 3 Cuerpo Académico (CA) 454 “Alimentación y Nutrición en el Proceso Salud-Enfermedad” Centro Universitario de Ciencias de la Salud, Universidad de Guadalajara, Guadalajara, Jalisco 44340, Mexico; 4 Facultad de Ciencias Químico-Biológicas, Universidad Autónoma de Guerrero, Chilpancingo de los Bravo, Guerrero 39087, Mexico; 5 Department of Physiology/Research Biomedical Institute of Murcia (IMIB)-Arrixaca, University of Murcia, Murcia 30120, Spain; 6 Laboratorio de Inmunología, Departamento de Fisiología, Centro Universitario de Ciencias de la Salud, Universidad de Guadalajara. Sierra Mojada 950, Guadalajara, Jalisco 44340, Mexico; 7 Servicio de Reumatología, O.P.D. Hospital Civil de Guadalajara Fray Antonio Alcalde, Guadalajara, Jalisco 44280, Mexico

**Keywords:** nutritional status, dietary status, systemic lupus erythematosus, excess weight, clinical activity, obesity, nutrients, deficiencies, immunomodulation

## Abstract

Obesity and nutrients intake deficiencies may contribute to the clinical manifestations and inflammatory processes in systemic lupus erythematosus (SLE). The aim of this study was to assess the relationship between nutritional status and dietary intake with clinical variables in Mexican-mestizo SLE patients. A cross-sectional study was conducted in 130 female SLE patients, classified by the 1997 SLE American College of Rheumatology (ACR) criteria; the clinical activity was evaluated by the Mexican-Systemic Lupus Erythematosus-Disease Activity Index (Mex-SLEDAI); body mass index (BMI) by the World Health Organization (WHO) criteria; the energy calculation and nutritional intake were performed by Nutritionist Pro Diet software. SLE patients with excess weight (BMI > 25 kg/m^2^) showed a higher score of clinical activity (Mex-SLEDAI = 2; *p* = 0.003), higher clinical activity prevalence (40.9%; *p* = 0.039) and a significant association for high clinical activity (odds ratio (OR) = 2.52; 95% confidence interval (CI) = 1.08–5.9; *p* = 0.033), in comparison with patients without excess weight (BMI < 25 kg/m^2^). In particular, the excess weight increased the Mex-SLEDAI score (β coefficient = 1.82; *R*^2^ = 0.05; *p* = 0.005). Also, the SLE patients presented a high prevalence (%) of deficient consumption (cut-off point: <67% of dietary adequacy) of vitamin E (100%), iodine (96%), omega 3 (93.44%), biotin (78%), vitamin K (73.33%), iron (67%), vitamin D (63.3%), potassium (59%), folic acid (56.67%), pantothenic acid (43.3%), vitamin A (41.67%) and zinc (32%). In conclusion, in SLE patients the excess weight was associated with increased clinical activity and to the presence of deficiencies in some essential nutrients ingested.

## 1. Introduction

Systemic lupus erythematosus (SLE) is the prototypic autoimmune disease with chronic inflammation, deposition of immune complexes and participation of autoreactive B and T cells with a predominance of Th2 inflammatory response [1,2,3]. The SLE incidence rate varies from 1 to 10 per 100,000 person/years, and the prevalence varies from 20 to 70 per 100,000 persons [4]. The disease mainly affects women in a 10:1 ratio [1], and the phenotypic expression varies among individuals from different populations, with a differential contribution of factors in the development and chronicity of SLE; such as ethnicity, gender and genetic susceptibility, and recently the presence of obesity and unhealthy dietary patterns have also been related to it [5,6,7].

An imbalance of nutritional status and daily dietary intake have been implicated as risk factors for exacerbation of clinical manifestations of several autoimmune diseases such as rheumatoid arthritis (RA) and SLE, through triggering pathways involved in the expression of the inflammatory cytokines such as tumor necrosis factor alpha (TNF-α) and interleukin 6 (IL-6) [7,8,9]. Previous studies conducted in different populations have reported that SLE patients might have a poor nutritional status and nutrients intake compared with the general population [6,7,10]. They usually have a high risk of low bone mineral density, deficiency in homocysteine and anemia; as well as more than a half of them have three or more risk factors for developing cardiovascular diseases, metabolic syndrome and type 2 diabetes mellitus; such as dyslipidemias, hypertension and obesity [11,12,13,14,15].

In particular, obesity could increase the levels of pro-inflammatory cytokines as TNF-α and IL-6 which can exacerbate the inflammatory process and increase the risk of higher mortality in SLE patients in comparison with healthy weight SLE patients [14,16,17,18,19]. Notably, nutritional status and the diet represent risk factors that can be modified with nutritional intervention, and prevent the development of obesity, cardiovascular disease and chronic systemic inflammation [5,7,18]. Concerning the diet, unhealthy dietary patterns such as Western diet have been typically associated with high levels of inflammation, while healthy diets such as the Mediterranean diet are associated with the induction of anti-inflammatory status [20,21].

In this context, the excessive consumption of calories, proteins, zinc, iron, refined grains, simple carbohydrates and saturated/trans fats can aggravate the symptoms of SLE and elevate C-reactive protein (CRP) and IL-6 serum levels [7]. Additionally, the high consumption of salt (NaCl) and high sodium serum levels have been described in vitro and in SLE prone murine models as favoring the differentiation of CD4+ Th17 helper cells (Th17 cells) [22]. Moreover, studies replicated in RA and SLE patients have also documented the role of sodium in the Th17 polarization, with potential implications in the clinical manifestations of the disease [23,24]. Therefore, these previous findings support the role of nutrients in the immune response modulation and highlight the potential contribution of an adequate nutritional and dietary status in the prognosis and development of comorbidities that modify the disease course and survival in SLE.

Based on this knowledge, we evaluated the relationship of the nutritional and dietary status with clinical variables in Mexican-Mestizo patients with systemic lupus erythematosus.

## 2. Materials and Methods

### 2.1. Subjects

A cross-sectional study was conducted in 130 female SLE patients from an unrelated Mexican-Mestizo population, classified according to the 1997 American College of Rheumatology criteria for SLE [25], recruited in 2017–2019 from the Rheumatology Department of the Hospital Civil Fray Antonio Alcalde, Guadalajara, Jalisco, Mexico.

The disease remission/activity presented at enrollment in the study was evaluated by the Mexican-Systemic Lupus Erythematosus-Disease Activity Index (Mex-SLEDAI) which is a validated activity index adjusted for the Mexican-Mestizo population [26], and the chronicity was evaluated by the Systemic Lupus International Collaborating Clinics (SLICC) criteria [27]. All SLE patients included in the present study were without recent infections, trauma nor surgery, pregnancy, neither other autoimmune systemic conditions not related to the SLE.

### 2.2. Ethical Considerations

All of the SLE patients before enrollment to the study provided signed written informed consent, and the protocol was approved by the Research Ethical Committee of the University of Guadalajara (CI-05018 CUCS-UdG), based on the ethical guidelines of the 2008 Declaration of Helsinki.

### 2.3. Anthropometric Measurements

Bodyweight and energy expenditure or fasting basal metabolic rate (BMR) were determined in the morning through the bioimpedance analysis prediction method, which is a validated formula confirmed by indirect calorimetry measurement described by the manufacturer (TANITA^®^ Ironman™ body composition Monitor BC-549, Arlington Heights, IL, USA), and height was measured to the nearest 0.1 cm using a stadiometer (Seca, Hamburg, Germany). From these measurements, the body mass index was calculated (BMI = weight/height^2^, kg/m^2^). Patients were classified as low weight (BMI < 18.5 kg/m^2^), healthy weight (BMI 18.6–24.9 kg/m^2^), overweight (BMI 25–29.9 kg/m^2^), or obese (BMI ≥ 30 kg/m^2^), and for the analysis we used the following BMI classification: a) SLE patients without excess weight (<25 kg/m^2^) and b) SLE patients with excess weight (>25 kg/m^2^) according to the NOM-043-SSA2-2012-MEX based on World Health Organization (WHO) criteria [28].

### 2.4. Biochemical Measurements and Definitions

A blood sample was obtained from each patient from antecubital venipuncture collected in the morning between 7:30 and 10:00 am after an overnight fast (12 h) and then centrifuged for 10 min to obtain serum. Total serum cholesterol, triglycerides, high density lipoprotein cholesterol (HDL-C), low density lipoprotein cholesterol (LDL-C) and glucose levels were obtained using semi-automated equipment (Mindray-BS-240 Clinical Chemistry Analyzer, Shenzhen, China) with colorimetric enzymatic assays using the BioSystem^®^ kits (Barcelona, Spain).

For the interpretation of serum lipid profile values, the guide for the treatment of dyslipidemias in adults was taken as reference from Adult Treatment Panel III (ATP III) [29]. Glucose was interpreted as: normal (<100 mg/dL), prediabetes (>100 and <125 mg/dL) and diabetes (>126 mg/dL), according to NOM-015-SSA2-2010-MEX, based on WHO criteria [30]. Systolic blood pressure (SBP) and diastolic blood pressure (DBP), were measured using a digital sphygmomanometer and classified according to the WHO-International Society of Hypertension Guidelines for the Management of Hypertension 1999 [31].

### 2.5. Nutritional Assessment

The evaluation of food consumption was realized in a personal interview carried out by a trained nutritionist-dietician by collecting three 24 h food records (two weekdays and one weekend day) and a semi-quantitative food frequency questionnaire was applied. Both are nutritional tools based on the validated questionnaires proposed by the 2016 Mexican National Health and Nutrition Survey (ENSANUT by its Spanish acronym) [32]. For a more accurate quantitative estimation of the food that the SLE patients ingested in each of their mealtimes, they were asked for quantity, type or variety and the additives used in the meals preparation, with the support of the “Mexican food photograph album” validated for the visual estimation of food in the Mexican population [33].

### 2.6. Energy, Nutritional Requirements and References Classification

The calculation of the energy and nutritional content of the collected dietary records was performed with the Nutritionist Pro Diet software (Axxya Systems, Washington, DC, USA). For each patient the energy requirement was calculated according to their individual total energy expenditure: BMR + 10% of the thermic effect of food + 10% of physical activity (sedentary). The energy distribution was calculated in percentages of each macronutrient considering its energy contribution: four calories per gram in the case of proteins and carbohydrates, and nine calories per gram in the case of lipids [34].

The adequate consumption of nutrients requirement of macronutrients was calculated using the average of the Mexican recommended distribution range ((min + max)/2) as a cut-off point: carbohydrates (59%), added sugars (<10%), proteins (17.5%), total fat (27.5%), saturated fat (<7%), monounsaturated fat (<15%), polyunsaturated fat (8%), trans fat (<1%), omega 6 (6.5%) and omega 3 (1.5%) [35].

Deficiencies or excess in the consumption of each dietary parameters were evaluated by their individual adequacy percentage (average nutrient consumption × 100/recommended daily average nutrient intake), the values of daily average nutrient intake were based on the following references: Dietary Reference Intakes (DRI) or Recommended Dietary Allowance (RDA) of the NOM-051-SCFI/SSA1-2010-MEX based on the recommendation of nutrient intake for Mexican population [34], the Institute of Medicine [36,37] and the WHO/Food and Agriculture Organization (FAO) [38], according to the nutrient evaluated.

The adequacy percentage values obtained were classified according to the interpretation proposed by Inano et al.: deficient (<67%), acceptable (≥67% and <90%), good (≥90% and ≤110%) and excessive (>110%) [39].

### 2.7. Statistical Analysis

The statistical analyses were performed with the software STATA v 9.2 (College Station, TX, USA), and GraphPad Prism v 5.0 (San Diego, CA, USA). The statistical power was evaluated according to the calculation of sample size, performed with an estimated error margin of 2% with a confidence degree of 95% and an expected prevalence of excess weight in SLE patients of 60% reported in previous studies [40]. The Shapiro–Wilk test was used to determine the nonparametric and parametric distribution of the continuous variables. For the descriptive analysis, the nominal discontinuous variables were expressed as frequencies; the continuous variables with parametric distribution were expressed as means ± standard deviation (SD) and the nonparametric variables as medians and percentiles 5–95.

For the inferential analysis, the *χ*^2^ test was used to compare proportions. For parametric quantitative determinations of two groups, the Student’s *t*-test was used, and the Mann–Whitney U test was used for nonparametric quantitative determinations. To evaluate the contribution of the excess weight to the clinical activity, we used models of linear and logistic regression. The differences were considered significant with a value of *p* < 0.05.

## 3. Results

A total of 130 female SLE patients were evaluated with a mean age of 40.6 ± 12.6 years old, of which 65.6% were in clinical remission (Mex-SLEDAI < 2) and 34.4% were in clinical activity (Mex-SLEDAI ≥ 2). The drugs with the highest prescription were glucocorticoids such as prednisone (57.7%) followed by chloroquine (51.5%) and hydroxychloroquine (44.3%). The overall SLE patients presented normal blood pressure median values, as well as blood biochemistry median values, such as glucose, total cholesterol, LDL-C and triglycerides, except for HDL-C, which was low with a median of 28.2 mg/dL (Table 1).

According to the BMI classification, the 70% of the SLE patients evaluated presented with excess weight (38.46% overweight and 31.54% obesity), while only 26.92% presented with healthy weight and 3.08% low weight (Table 1).

When comparing the continuous variable of the Mex-SLEDAI by the BMI classification, we observed significant differences in the clinical activity score values: (a) low weight (Mex-SLEDAI = 0 (0–0)), (b) healthy weight (Mex-SLEDAI = 0 (0–6)), (c) overweight (Mex-SLEDAI = 2 (0–10)) and (d) obese (Mex-SLEDAI= 2 (0–10)) (*p* = 0.008), with a similar clinical activity score in overweight and obese SLE patients. Moreover, the BMI had a low positive correlation with the Mex-SLEDAI index score (Spearman’s rho = 0.27, *p* = 0.036) (data not shown).

Concerning the clinical characteristics stratified according to BMI, and due to the similar values of Mex-SLEDAI index score presented by overweight and obese SLE patients, we decided to group the patients in with and without excess weight. According to these two subgroups of the BMI, significant differences were observed in the clinical activity evaluated by the Mex-SLEDAI index score. SLE patients with excess weight showed a higher score of clinical activity in comparison with SLE patients without excess weight, who showed a median of clinical activity in the remission range (Mex-SLEDAI: BMI < 25 kg/m^2^ = 0 vs. BMI > 25 kg/m^2^ = 2; *p* = 0.003) (Table 2). Following this stratification, a higher prevalence of clinical activity (Mex-SLEDAI ≥ 2) was observed in the subgroup with excess weight (BMI < 25 kg/m^2^ = 21.6% vs. BMI > 25 kg/m^2^ = 40.9%; *p* = 0.039) (Table 2).

To estimate the contribution of excess weight to the clinical activity evaluated by the Mex-SLEDAI index, multiple logistic and linear regression models were used. Notably, we found that excess weight in SLE patients was associated with a significant increase of 2.52 fold for a higher clinical activity in comparison with SLE patients without excess weight (BMI > 25 kg/m^2^: odds ratio (OR) = 2.52; 95% confidence interval (CI) = 1.08–5.9; *p* = 0.033), and the excess weight also contributed to a significant increase to the clinical activity Mex-SLEDAI score (β coefficient = 1.82; *R*^2^ = 0.05; *p* = 0.005), highlighting the relationship of excess weight with the clinical activity in the SLE patients evaluated (data not shown).

Moreover, in this same subgroup of SLE patients with excess weight, high values were observed within the normal range of systolic (*p* = 0.028) and diastolic blood pressure (*p* = 0.043) with a median of 110/71.5 mmHg. When we evaluated the cardiometabolic risk according to the proposed stratification by BMI, the same pattern of biochemical alterations was observed in the same subgroup of SLE patients, which showed significant differences in glucose levels (*p* = 0.045) in conjunction with a higher prevalence of alterations in glycemia (*p* = 0.006), of which 20.5% had prediabetes (100–125 mg/dL) and 8.4% type 2 diabetes mellitus (>126 mg/dL) compared to patients without excess weight, where 97.1% of patients presented normal glucose values (<100 mg/dL) (Table 2).

Regarding the lipid profile, the SLE patients with excess weight had significantly higher values of triglycerides (BMI < 25 kg/m^2^ = 98.42 mg/dL vs. BMI > 25 kg/m^2^ = 125.7 mg/dL; *p* = 0.0007) and lower HDL-C values (BMI < 25 kg/m^2^ = 38.2 mg/dL vs. BMI > 25 kg/m^2^ = 25.8 mg/dL; *p* = 0.0009) compared to the group of SLE patients without excess weight (Table 2).

In order to evaluate the nutrimental dietary content of the foods consumed by all SLE patients, we found that the overall of SLE patients presented an average consumption of 1543 calories, with a distribution of 53.5% of carbohydrates, 4.2% of added sugars, 16.5% of proteins, 30.2% of total fat, 8.3% of monounsaturated fat and 0.3% of trans fats, which were found within the distribution percentages recommended for the Mexican population [34]. However, they had a lower mean consumption than the recommended intake of essential fatty acids, such as omega 3 (0.57 g/day) and omega 6 (7.37 g/day) as well as polyunsaturated fat (9.4 g/day) and vitamins such as vitamin A (485.5 μg RE/day), vitamin D (3 μg/day), vitamin E (0.9 mg/day) and vitamin K (33.2 μg/day) and conversely, a higher consumption than recommended of saturated fat (14.5 g/day), minerals, such as sodium (1695.7 mg/day), phosphorus (1109.2 mg/day), magnesium (292 mg/day), selenium (67.7 μg/day) and vitamins, such as vitamin C (111.4 mg/day) (Table 3).

**Figure 1:**
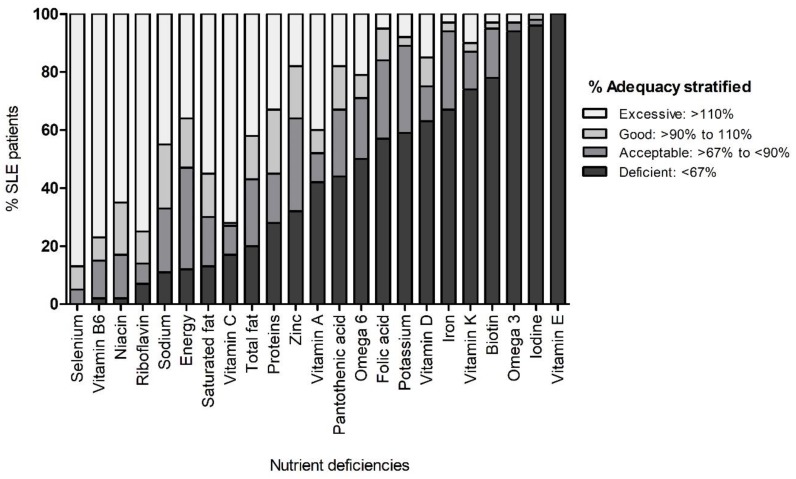
Intake of systemic lupus erythematosus patients according to their most common nutrient deficiencies. Data provided in percentages.

Taking as reference the DRI/RDA of vitamins and minerals and the nutritional distribution percentages of macronutrients suggested for the Mexican population, we stratified the nutrimental intake of all SLE patients according to the cut-off values of the dietary adequacy percentage [39]. We observed a high prevalence of deficient consumption (<67% of dietary adequacy percentage) of several nutrients in the SLE patients evaluated, mainly for: vitamin E (100%), iodine (96%), omega 3 fatty acids (93.44%), biotin (78%), vitamin K (73.33%), iron (67%), vitamin D (63.3%), potassium (59%), folic acid (56.67%), pantothenic acid (43.3%), vitamin A (41.67%) and zinc (32%) (Figure 1). Likewise, other nutrients evaluated showed a high prevalence of excessive consumption (>110% of dietary adequacy percentage) such as selenium (87%), vitamin B6 (77%), riboflavin (75%), vitamin C (72%), niacin (65%), saturated fat (55%) and sodium (45%) (Figure 1).

When the dietary intake of SLE patients was compared according to the BMI subgroup proposed in this study, a greater nutrimental consumption of all the nutrients was found in the patients without excess weight, with a significant difference in terms of energy (*p* = 0.009), proteins (*p* = 0.012), total fat (*p* = 023), saturated fat (*p* = 0.015), monounsaturated fat (*p* = 0.033), cholesterol (*p* = 0.045), sodium (*p* = 0.041), potassium (*p* = 0.016), as well as a higher consumption of minerals, such as zinc (*p* = 0.025), selenium (*p* = 0.007), iodine (*p* = 0.021) and B vitamins, such as riboflavin (*p* = 0.039), niacin (*p* = 0.016), vitamin B6 (*p* = 0.017) and pantothenic acid (*p* = 0.029) compared to SLE patients with excess weight (Table 4).

## 4. Discussion

Autoimmune diseases have increased their incidence and prevalence in Western countries. The pathogenesis of SLE is complex and multifactorial, with an interaction of genetic and environmental factors involved in its pathophysiology. Various environmental factors modulate the expression of the disease clinical manifestations; within these, dietary habits such as excessive salt intake, processed foods, vitamin D deficiency and the presence of obesity have also been implicated in modifying the severity of the course of autoimmune diseases [5,6,9,10,11,12,14,16,18,24,41,42].

In the present study, we found that SLE patients with excess weight (BMI > 25 kg/m^2^) showed a higher score of clinical activity, higher clinical activity prevalence and significant association to present higher clinical activity scores in comparison with patients without excess weight. In particular, in our study, we found that excess weight was related to a higher SLE clinical activity score evaluated by the Mex-SLEDAI index.

Previous studies conducted in other populations of SLE patients supported these findings in our study; it has been reported that SLE patients present a high frequency of overweight and obesity, and the prevalence of excess weight (BMI > 25 kg/m^2^) in the majority of studies conducted in SLE patients varies from 56 to 67% [10,11,14,16,17]. In Brazilian SLE patients two studies evaluating their nutritional status, reported a prevalence of excess weight from 62.4% to 64.1% [6,11], and in Spanish SLE patients the prevalence reported is of 43.5% with the presence of nutritional deficiencies [43]. Another study reported that 67% of SLE Polish patients are with excess weight, and carry higher concentrations of inflammatory markers such as C-reactive protein (CRP), in comparison with normal-weight patients [44]. These previous findings reported highlight the potential contribution of excess weight in the modulation of the inflammatory process of SLE.

Currently, the presence of obesity has emerged as an important factor that could modify the severity and chronicity of the autoimmune pathologies, through the secretion of pro-inflammatory cytokines involved in the onset and progression of several autoimmune diseases, including RA, multiple sclerosis, psoriasis and psoriatic arthritis [18].

Notably, it has been reported that obese SLE patients have increased gene and protein expression of several pro-inflammatory cytokines such as IL-23 [45] and TNF-α, associated with total fat mass in pediatric SLE patients [46]. This inflammation status associated with obesity creates a background with an increased likelihood to develop complications such as metabolic syndrome in SLE patients [47,48]. In experimental studies, it has been suggested that high levels of adipocytokines such as leptin could be related to the development of autoimmunity in obese SLE-prone mice [49,50].

Regarding the clinical activity, a high BMI appears in several clinical studies to be associated with more severe cognitive and renal involvement [13,44,51], which are evaluated within the clinical activity criteria for SLE. In particular, in a study conducted in a multi-ethnic cohort of SLE patients by Teh et al. [17], showed that an increased BMI was associated with worse disease activity evaluated by the SLEDAI [17]. Therefore, an increased adiposity and a high prevalence of obesity in SLE could contribute to the worsening of its chronic inflammatory status, increasing the oxidative stress with a secondary inflammation sustained in a feedback loop of oxidative stress through the increase of pro-inflammatory cytokines and acute-phase reactants [18].

In relation with the cardiometabolic variables evaluated according to the stratification by BMI, the same pattern of biochemical alterations was observed in the subgroup of SLE patients with excess weight, which showed significant differences in glucose levels in conjunction with a higher prevalence of alterations in glycemia, such as prediabetes and type 2 diabetes mellitus, in comparison with SLE patients without excess weight where patients presented normal glucose values. Regarding the lipid profile, the SLE patients with excess weight had significantly higher values of triglycerides and significantly lower HDL-C values, compared to the group of SLE patients without excess weight.

These findings found in our study, could be explained in part by previous findings reported in other studies where the abnormalities in plasma lipid concentrations are common in SLE patients [52], and the prevalence of dyslipidemias with an increase in total cholesterol, LDL-C, triglycerides and reduction in HDL-C has been reported in about 30% of patients with a recent diagnosis of SLE; and after three years of diagnosis, dyslipidemias reach a prevalence of 60% [53]. These conditions have been associated with a bimodal pattern of mortality in SLE patients, which has an initial peak as a result of disease activity and a late peak attributable to the development of atherosclerosis [15,54].

High levels of LDL-C, triglycerides and low levels of HDL-C are recognized and described as a “lupus pattern of dyslipoproteinemia” in patients with SLE [15]. During the active course of the disease, soluble lipid levels increase significantly compared to patients in remission, and correlates positively with the increase in clinical activity index scores [15], which could suggest that an abnormal lipid profile coupled with the presence of obesity is related to the activity of the disease.

Therefore, the presence of obesity is an essential trigger for these metabolic alterations in SLE patients. A positive energy balance in the metabolic status is associated with the development of obesity, which highly depends on diet and can favor or not the development of immune abnormalities manifesting with varied symptoms in organs involved in an interaction with genetic and environmental factors related to autoimmunity [18].

Dietary habits directly correlate with body weight, and a dietetic restriction of macronutrients or/and calorie restriction have been described to be beneficial in reducing disease manifestations in SLE-prone mice [50,55], including a lowered atherogenic risk, through mechanisms that include the induction of immunosuppressive Tregs cells [50] and reduction of expression of IL-12 and interferon gamma (IFN-γ), the nuclear factor kappa-light-chain-enhancer of activated B cells (NF-кB) as well as the 5′ AMP-activated protein kinase (AMPK) activation and the mammalian target of rapamycin (mTOR) signaling inactivation; both pathways known to be necessary for nutrient sensing and orchestrating the metabolic “switches” for immune cell activation [55,56,57].

In regard to this, we found that the SLE patients had a lower consumption than the recommended intake of nutrients such as essential fatty: omega 3, omega 6 and polyunsaturated fat as well as of vitamins, such as vitamin A, vitamin D, vitamin K and vitamin E and a higher consumption than recommended of saturated fat, minerals such as sodium, phosphorus, magnesium, selenium and vitamins such as vitamin C.

Concerning the low consumption of essential fatty acids found in our study, in Swedish SLE patients a decreased intake of omega 3 and omega 6 compared to healthy subjects, and the content in adipose tissue of omega 3 was negatively associated with disease activity has been reported [12]. The results in another study in SLE patients from Northern Ireland showed that the supplementation with omega 3 fish oils were found to reduce symptomatic disease activity [58]. The immune cells involved in the inflammatory response are typically rich in omega 6 and arachidonic acid, which is a precursor to inflammatory eicosanoids such as prostaglandins, thromboxanes and leukotrienes, which are mediators and regulators of the inflammatory cascade, immunity and platelet aggregation [59].

Regarding the low consumption of vitamins described in our study, low levels of vitamin D have been described as a common characteristic in SLE patients associated with increased disease activity [47]. Low levels of serum vitamin D are associated with avoidance of sun exposure, reduced dietary intake and the presence of polymorphisms related to the metabolism of vitamin D, as in its receptor expression and hepatic cytochromes [60]. Vitamin D supplementation in SLE has been associated with increased numbers of Tregs cells [61] and the inhibition of the formation of neutrophil extracellular traps that causes endothelial damage and the expression of inflammatory cytokines in SLE [42].

About vitamin A, its metabolites such as retinoic acid, show that retinoids inhibit the formation of pro-inflammatory Th17 cells and promote the production of Tregs cells in SLE-prone mice, and in SLE patients improved their proteinuria, their high levels of anti-double stranded DNA antibodies (anti-dsDNA), and low titers of complements [62]. Also it is noteworthy that in our study we observed a high prevalence of deficiency in vitamin E intake. In previous studies it has been reported that the combination of fish oil (omega 3 and 6) with vitamin E has an impact on several SLE mediators, mice fed with fish oil and vitamin E showed a reduction in leukotriene, thromboxane and immunomodulatory cytokines such as IL-6, IL-10, IL-12 and TNF-α [7].

Our findings related to nutrient consumption deficiencies in SLE patients have a similar pattern to that reported in a study conducted by Pocovi-Gerardino et al. [43], where SLE patients did not reach the recommended intake for the Spanish population for iron (88%), calcium (65.2%), iodine (92.4%), potassium (73.9%), magnesium (65%), folate (72.8%) and vitamins E (87%) and D (82.6%) [43], and in a similar way to our findings, the SLE patients exceeded the recommendations for phosphorus and sodium.

The role of dietary sodium on immune functions has been explored in several murine models and humans [22,23,24,63]. Studies showed that an excess salt intake could modulate T cells immune response, and high concentrations of NaCl promote the differentiation of Th cells toward the pro-inflammatory Th17 phenotype via the serum- and glucocorticoid-induced kinase 1 (SGK1) mediator [22,64], related to an essential role in controlling the balance between Treg and Th17 cells.

Following these reported findings, we found that deficiencies and excess consumption of the nutrients evaluated were more evident when we stratified the nutrimental intake of all SLE patients according to the cut-off values of the dietary adequacy percentage proposed by Inano et al. [39]. When dietary intake of SLE patients was compared according to the presence or absence of excess weight, a higher nutrimental consumption of all the nutrients was found in the patients without excess weight, but with a significant difference in terms of energy, macronutrient distribution as well as a higher consumption of minerals such as zinc, selenium, iodine and B vitamins. Nevertheless, they had an excess in the consumption of sodium and potassium, in comparison with SLE patients with excess weight.

One explanation for these findings for the difference in nutritional consumption, may be that a protective factor for the SLE patients without excess weight was that they had a higher consumption of immunomodulatory minerals and vitamins such as selenium, vitamin C and B vitamins in comparison with patients with excess weight; these minerals and vitamins are known to protect against tissue damage by activation of macrophages, monocytes and granulocytes and suppression of the activity of TNF-α, the main cytokine related to damage in SLE [7].

Therefore, these findings evidence that the dietary habits of SLE patients in several studies have a common pattern with low intake of immunomodulatory nutrients such as omega 3, omega 6, fiber and vitamins A, D and E, as well as a high consumption of saturated fats and sodium. By this way, it suggests that a balanced diet including a moderate calorie restriction from macronutrients such as proteins and carbohydrates with sufficient sources of mono and polyunsaturated fatty acids, vitamins and minerals with antioxidants effects, could protect against tissue damage and suppress inflammatory activity in SLE.

The constraint of the present study was that our cross-sectional study design limited us by simply showing an association between excess weight and Mex-SLEDAI but we do not suggest causality, because it only provides information of a specific point in time. Moreover the limitations of the present study were that we did not quantify the serum levels of the nutrients evaluated through nutrient intake, and consumption deficiencies reported may not be represented in their blood serum levels, because the dietary records were subject to memory errors and may not have represented at all the routine intake of the patients evaluated.

Further prospective studies in a SLE Mexican population cohort are needed to be performed, in order to evaluate causality in the relationship between obesity and clinical activity described in this cross-sectional study. Moreover, functional studies focused on assessing the potential role in the immunomodulation of serum nutrient levels in an autoimmune context will be necessary to perform to clarify the associations found in relation to nutritional and dietary status in SLE patients. Both future studies will help to support the nutritional interventions in subsequent studies conducted in patients with autoimmune diseases.

## 5. Conclusions

In conclusion, our findings show that in SLE patients the excess weight was associated with an increase in the clinical activity and with the presence of deficiencies in some essential immunomodulatory nutrients ingested such as omega 3, omega 6 and vitamins A, D, K and E.

## Figures and Tables

**Table 1:** Clinical characteristics and nutritional status from overall systemic lupus erythematosus patients.

Variable	Value
**Age (years) ^a^**	40.6 ± 12.6
**Time of evolution (years) ^b^**	7.6 (1–22)
**SLICC ^b^**	0 (0–4)
**Mex-SLEDAI ^b^**	0 (0–8)
Remission (<2) ^c^	65.6 (84/128)
Activity (≥2) ^c^	34.4 (43/128)
**Antibodies (positive)**	
Antinuclear (ANAs) ^c^	76.7 (89/116)
Anti-double stranded DNA (Anti-dsDNA) ^c^	73.5 (25/34)
Anti-La ^c^	13.3 (2/15)
Anti-Ro ^c^	12.5 (2/16)
**Treatment prevalence% (n)**	
Prednisone ^c^	57.7 (75/130)
Chloroquine ^c^	51.5 (67/130)
Hydroxychloroquine ^c^	44.3 (27/61)
Azathioprine ^c^	39.2 (51/130)
Mycophenolic acid ^c^	30.8 (40/130)
**Clinical chemistry**	
Glucose (mg/dL) ^b^	88 (70.4–130.3)
Total Cholesterol (mg/dL) ^b^	168.7 (113.4–249.5)
Triglycerides (mg/dL) ^b^	122.7 (51.1–245.8)
LDL-C (mg/dL) ^b^	73.8 (45.9–128.7)
HDL-C (mg/dL) ^b^	28.2 (13.1–61.7)
**Blood pressure**	
Systolic (mmHg) ^b^	111 (90–141)
Diastolic (mmHg) ^b^	74 (60–91)
**Nutritional status**	
Height (cm) ^a^	158 ± 0.06
Weight (kg) ^b^	67.6 (50.4–95.9)
BMI (kg/m^2^) ^a^	27.9 ± 5.34
**BMI classification**	
Low weight (<18.5) ^c^	3.08 (4/130)
Normal weight (18.5–24.9) ^c^	26.92 (35/130)
Overweight (25–29.9) ^c^	38.46 (50/130)
Obese (>30) ^c^	31.54 (41/130)

^a^ Data provided in average ± standard deviation. ^b^ Data provided in median (percentile: p5th–p95th). ^c^ Data provided in percentages (n/total patients evaluated).

**Table 2:** Clinical and biochemical characteristics of the systemic lupus erythematosus stratified according to the body mass index (BMI).

Variable	BMI < 25 kg/m^2^ (*n* = 39)	BMI > 25 kg/m^2^ (*n* = 91)	*p* Value
**Age (years) ^a^**	35.10 ± 1.72	42.26 ± 1.30	0.999
**Mex-SLEDAI ^c^**	0 (0–6)	2 (0–10)	**0.003**
Mex-SLEDAI % (n)			**0.039**
Remission (<2) ^b^	78.4 (29/37)	59.1 (52/88)	
Activity (≥2) ^b^	21.6 (8/37)	40.9 (36/88)	
**Blood pressure**			
Systolic (mmHg) ^c^	110 (90–139)	113 (100–143)	**0.028**
Diastolic (mmHg) ^c^	71.5 (60–90)	77 (64–97)	**0.043**
**Blood pressure (SBP/DBP mmHg) % (n)**			0.354
Normal (<130/<85) ^b^	82.1 (23/28)	61.3 (19/31)	
Normal high (130–139/85–89) ^b^	10.7 (3/28)	16.1 (5/31)	
AH systolic (140–149/<90) ^b^	7.1 (2/28)	12.9 (4/31)	
AH grade 1 (140–159/90–99) ^b^	0 (0/28)	6.4 (2/31)	
AH grade 2 (160–179/100–109) ^b^	0 (0/28)	0 (0/31)	
AH grade 3 (≥180/≥110) ^b^	0 (0/28)	3.2 (1/31)	
**Glucose (mg/dL)**	85.55 (65.82–98.76)	90.08 (70.4–132.95)	**0.045**
Glucose classification (% n)			**0.006**
Normal (<100 mg/dL) ^b^	97.1 (34/35)	71.1 (59/83)	
Prediabetes (100–125 mg/dL) ^b^	2.9 (1/35)	20.5 (17/83)	
Diabetes (>126 mg/dL) ^b^	0 (0/35)	8.4 (7/83)	
**Lipid profile**			
Triglycerides (mg/dL) ^c^	98.42 (45.5–168.6)	125.7 (57.7–260.6)	**0.0007**
Total Cholesterol (mg/dL )^c^	159.5 (112–240.7)	175 (117.02–250.8)	0.077
LDL-C (mg/dL) ^c^	79.74 (47.85–128.7)	72.4 (41.3–130.7)	0.215
HDL-C (mg/dL) ^c^	38.2 (15.94–66.7)	25.8 (12.8–54.4)	**0.0009**

^a^ Data provided in average ± standard deviation, Student’s *t*-test. ^b^ Data provided in percentages (n/total patients evaluated), *χ*^2^ test. ^c^ Data provided in median (percentile: p5th–p95th), Mann–Whitney test. AH: arterial hypertension. The bold letters indicate the variables with significant differences.

**Table 3:** Intake of energy and nutrients in all systemic lupus erythematosus (SLE) patients and recommended intake.

Nutrient	Intake (U/day)	Recommended Intake *
**Energy (calories) ^a^**	1543 ± 407	1563 (1358–1963)
**Dietary fiber (g) ^b^**	21.6 (10.7–39.9)	30
**Macronutrients**		
Carbohydrates (g) ^a^	204 ± 55.5	230 (200–289)
Added sugars (g) ^a^	14.8 (0.53–59.7)	38.2 ± 10
Proteins (g) ^b^	62.8 (37.6–105)	68.3 (59.4–85.9)
Total fat (g) ^b^	45.5 (28.3–95.7)	47.7 (41.5–59.9)
Saturated (g) ^b^	14.5 (7.02–26.6)	11.9 (10.4–15)
Monounsaturated (g) ^b^	13.7 (8.20–30.1)	25.9 (22.5–32.5)
Polyunsaturated (g) ^b^	9.4 (4.81–19.9)	13.9 (12.1–17.4)
Trans (g) ^b^	0.47 (0.05–0.99)	1.72 (1.49–2.16)
Cholesterol (mg) ^b^	157.7 (59.7–373.6)	<300 ****
Omega 3 fatty acids (g) ^b^	0.57 (0.33–2.38)	2.6 (2.26–3.27)
Omega 6 fatty acids (g) ^b^	7.37 (4.04–17.3)	11.3 (9.81–14.2)
**Vitamins**		
Vitamin A (μg RE) ^b^	485.5 (123.7–2057)	568
Vitamin C (mg) ^b^	111.4 (12.3–324.9)	60
Vitamin D (μg) ^b^	3 (0.38–9.36)	5.6
Vitamin E (mg) ^b^	0.9 (0.04–3.83)	11
Thiamin (mg) ^a^	1.10 ± 0.39	0.8
Riboflavin (mg) ^b^	1.14 (0.52–2.38)	0.84
Niacin (mg) ^b^	13.9 (7.87–23.3)	11
Vitamin B6 (mg) ^b^	1.5 (0.65–2.48)	0.93
Folic acid (μg) ^b^	228.2 (111.7–426.8)	380
Vitamin B12 (μg) ^b^	2.55 (1.04–6.77)	2.1
Biotin (μg) ^b^	11.5 (2.14–28.7)	30 ***
Pantothenic acid (mg) ^b^	2.91 (1.26–5.96)	4
Vitamin K (μg) ^b^	33.2 (12.1–149.9)	78
**Minerals**		
Sodium (mg) ^b^	1695.7 (913.8–3595.7)	1600 **
Potassium (mg) ^b^	2210.4 (1079.7–3909.6)	3500 ***
Calcium (mg) ^b^	740.7 (346.5–1485.8)	900
Phosphorus (mg) ^b^	1109.2 (674.4–1989.4)	664
Iron (mg) ^b^	10 (6.33–16.2)	17
Magnesium (mg) ^a^	292 ± 93.6	248
Zinc (mg) ^a^	8.5 ± 2.75	10
Selenium (µg) ^b^	67.7 (34.4–123.1)	41
Copper (mg) ^b^	1.20 (0.63–2.18)	0.65
Iodine (μg) ^b^	0.6 (0–61.2)	99

^a^ Data provided in average ± standard deviation. ^b^ Data provided in median (percentile: p5th–p95th). RE: retinol equivalents. * Dietary Reference Intakes (DRI) or Recommended Dietary Allowance (RDA) [34]; ** recommendation of nutrient intake for Mexican population [34]; *** [36,37] **** [38].

**Table 4:** Nutrient intake stratified according to the BMI of systemic lupus erythematosus patients.

Variable	BMI < 25 kg/m^2^ (*n* = 39)	BMI > 25 kg/m^2^ (*n* = 91)	*p* Value
**Energy (calories) ^a^**	1672.6 ± 73.24	1429.9 ± 69.8	**0.009**
**Dietary fiber (g) ^b^**	22.54 (11.9–34.9)	19.6 (10.4–43.5)	0.230
**Macronutrients**			
Carbohydrates (g) ^a^	212.3 ± 9.65	197.15±10.43	0.147
Added sugars (g) ^a^	15.4 (0–50.6)	14.7 (1.06–90.9)	0.289
Proteins (g) ^b^	72.2 (38.1–107.2)	52.9 (31.7–98.8)	**0.012**
Total fat (g) ^b^	52.9 (27.5–115.3)	41.1 (29.1–87.0)	**0.023**
Saturated (g) ^b^	18.2 (7.12–30.7)	11.9 (6.92–25.0)	**0.015**
Monounsaturated (g) ^b^	15.7 (8.72–31.1)	12.2 (8.14–28.9)	**0.033**
Polyunsaturated (g) ^b^	10.7 (4.71–26.3)	8.40 (4.91–18.8)	0.112
Trans (g) ^b^	0.49 (0.07–0.97)	0.42 (0.03–1.84)	0.882
Cholesterol (mg) ^b^	192.6 (52.9–414.6)	135.1 (60.9–271.2)	**0.045**
Omega 3 fatty acids (g) ^b^	0.68 (0.39–1.32)	0.57 (0.33–2.53)	0.292
Omega 6 fatty acids (g) ^b^	8.69 (2.49–21.57)	6.71 (3.97–17.0)	0.153
**Vitamins**			
Vitamin A (μg RE) ^b^	566.8(199.2–1455.9)	371.5 (57.5–2170.6)	0.486
Vitamin C (mg) ^b^	121.5 (36.3–253.4)	90.36 (4.35–345.1)	0.254
Vitamin D (μg) ^b^	3.61 (0.92–9.28)	2.83 (0.33–9.44)	0.407
Vitamin E (mg) ^b^	0.94 (0.04–3.71)	0.87 (0.05–4.59)	0.824
Thiamin (mg) ^a^	1.17 ± 0.06	1.03 ± 0.07	0.093
Riboflavin (mg) ^b^	1.21 (0.85–2.44)	1.04 (0.48–2.33)	**0.039**
Niacin (mg) ^b^	14.9 (8.51–21.1)	12.5 (7.66–23.5)	**0.016**
Vitamin B6 (mg) ^b^	1.66 (0.77–2.30)	1.22 (0.63–2.53)	**0.017**
Folic acid (μg) ^b^	257.1 (127.5–415.9)	219.1 (74.3–437.7)	0.182
Vitamin B12 (μg) ^b^	2.80 (1.04–5.66)	2.28 (1.05–8.94)	0.123
Biotin (μg) ^b^	13.1 (2.35–35.7)	9.34 (2.10–25.7)	0.230
Pantothenic acid (mg) ^b^	3.27 (1.24–5.93)	2.51 (1.28–5.99)	**0.029**
Vitamin K (μg) ^b^	36.8 (13.2–130.6)	30.6 (11.1–169.6)	0.614
**Minerals**			
Sodium (mg) ^b^	2005 (1113–4606)	1603 (773–3449)	**0.041**
Potassium (mg) ^b^	2414 (1117–3921)	1912 (1055–3899)	**0.016**
Calcium (mg) ^b^	750 (365–1469)	669 (332–1502)	0.213
Phosphorus (mg) ^b^	1228 (680–1946)	1049 (669–2033)	0.086
Iron (mg) ^b^	10.4 (7.12–15.6)	9.42 (5.77–16.7)	0.286
Magnesium (mg) ^a^	309.20 ± 17.6	276.9 ± 16.4	0.093
Zinc (mg) ^a^	9.23 ± 0.54	7.84 ± 0.44	**0.025**
Selenium (µg) ^b^	75.7 (39.9–152.5)	63.3 (31.4–103.8)	**0.007**
Copper (mg) ^b^	1.27 (0.69–1.74)	1.01 (0.57–2.65)	0.208
Iodine (μg) ^b^	1.19 (0–84)	0 (0–11.7)	**0.021**

^a^ Data provided in average ± standard deviation, Student’s *t*-test. ^b^ Data provided in median (percentile: p5th-p95th), Mann–Whitney test. The bold letters indicate the variables with significant differences.

## References

[1] Manson J.J., Rahman A. (2006). Systemic lupus erythematosus. Orphanet J. Rare Dis..

[2] Tsokos G.C. (2011). Systemic lupus erythematosus. N. Engl. J. Med..

[3] Rahman A., Isenberg D.A. (2008). Systemic lupus erythematosus. N. Engl. J. Med..

[4] Pons-Estel G.J., Alarcón G.S., Scofield L., Reinlib L., Cooper G.S. (2010). Understanding the epidemiology and progression of systemic lupus erythematosus. Semin. Arthritis Rheum..

[5] Aparicio-Soto M., Sánchez-Hidalgo M., Alarcón-de-la-Lastra C. (2017). An update on diet and nutritional factors in systemic lupus erythematosus management. Nutr. Res. Rev..

[6] Borges M.C., dos Santos F.D.M.M., Telles R.W., Lanna C.C.D., Correia M.I.T.D. (2012). Nutritional status and food intake in patients with systemic lupus erythematosus. Nutrition.

[7] Klack K., Bonfa E., Borba Neto E.F. (2012). Diet and nutritional aspects in systemic lupus erythematosus. Rev. Bras. Reumatol..

[8] Postal M., Appenzeller S. (2011). The role of Tumor Necrosis Factor-alpha (TNF-α) in the pathogenesis of systemic lupus erythematosus. Cytokine.

[9] Cutolo M., Nikiphorou E. (2018). Don’t neglect nutrition in rheumatoid arthritis!. RMD Open.

[10] Dos Santos F.D.M.M., Borges M.C., Correia M.I.T.D., Telles R.W., Lanna C.C.D. (2010). Assessment of nutritional status and physical activity in systemic lupus erythematosus patients. Rev. Bras. Reumatol..

[11] De Miranda Moura dos Santos F., Borges M.C., Telles R.W., Correia M.I.T.D., Lanna C.C.D. (2013). Excess weight and associated risk factors in patients with systemic lupus erythematosus. Rheumatol. Int..

[12] Elkan A.-C., Anania C., Gustafsson T., Jogestrand T., Hafström I., Frostegård J. (2012). Diet and fatty acid pattern among patients with SLE: Associations with disease activity, blood lipids and atherosclerosis. Lupus.

[13] Katz P., Yazdany J., Julian L., Trupin L., Margaretten M., Yelin E., Criswell L.A. (2011). Impact of obesity on functioning among women with systemic lupus erythematosus. Arthritis Care Res..

[14] Rizk A., Gheita T.A., Nassef S., Abdallah A. (2012). The impact of obesity in systemic lupus erythematosus on disease parameters, quality of life, functional capacity and the risk of atherosclerosis: Obesity in SLE. Int. J. Rheum. Dis..

[15] Szabó M.Z., Szodoray P., Kiss E. (2017). Dyslipidemia in systemic lupus erythematosus. Immunol. Res..

[16] Tedeschi S.K., Barbhaiya M., Malspeis S., Lu B., Sparks J.A., Karlson E.W., Willett W., Costenbader K.H. (2017). Obesity and the risk of systemic lupus erythematosus among women in the Nurses’ Health Studies. Semin. Arthritis Rheum..

[17] Teh P., Zakhary B., Sandhu V.K. (2019). The impact of obesity on SLE disease activity: Findings from the Southern California Lupus Registry (SCOLR). Clin. Rheumatol..

[18] Versini M., Jeandel P.-Y., Rosenthal E., Shoenfeld Y. (2014). Obesity in autoimmune diseases: Not a passive bystander. Autoimmun. Rev..

[19] Weisberg S.P., McCann D., Desai M., Rosenbaum M., Leibel R.L., Ferrante A.W. (2003). Obesity is associated with macrophage accumulation in adipose tissue. J. Clin. Investig..

[20] Shivappa N., Steck S.E., Hurley T.G., Hussey J.R., Hébert J.R. (2014). Designing and developing a literature-derived, population-based dietary inflammatory index. Public Health Nutr..

[21] Ruiz-Canela M., Zazpe I., Shivappa N., Hébert J.R., Sánchez-Tainta A., Corella D., Salas-Salvadó J., Fitó M., Lamuela-Raventós R.M., Rekondo J. (2015). Dietary inflammatory index and anthropometric measures of obesity in a population sample at high cardiovascular risk from the PREDIMED (PREvención con DIeta MEDiterránea) trial. Br. J. Nutr..

[22] Kleinewietfeld M., Manzel A., Titze J., Kvakan H., Yosef N., Linker R.A., Muller D.N., Hafler D.A. (2013). Sodium chloride drives autoimmune disease by the induction of pathogenic TH17 cells. Nature.

[23] Scrivo R., Massaro L., Barbati C., Vomero M., Ceccarelli F., Spinelli F.R., Riccieri V., Spagnoli A., Alessandri C., Desideri G. (2017). The role of dietary sodium intake on the modulation of T helper 17 cells and regulatory T cells in patients with rheumatoid arthritis and systemic lupus erythematosus. PLoS ONE.

[24] Scrivo R., Perricone C., Altobelli A., Castellani C., Tinti L., Conti F., Valesini G. (2019). Dietary Habits Bursting into the Complex Pathogenesis of Autoimmune Diseases: The Emerging Role of Salt from Experimental and Clinical Studies. Nutrients.

[25] Hochberg M.C. (1997). Updating the American college of rheumatology revised criteria for the classification of systemic lupus erythematosus. Arthritis Rheum..

[26] Uribe A.G., Vilá L.M., McGwin G., Sanchez M.L., Reveille J.D., Alarcón G.S. (2004). The Systemic Lupus Activity Measure-revised, the Mexican Systemic Lupus Erythematosus Disease Activity Index (SLEDAI), and a modified SLEDAI-2K are adequate instruments to measure disease activity in systemic lupus erythematosus. J. Rheumatol..

[27] Gladman D., Ginzler E., Goldsmith C., Fortin P., Liang M., Urowitz M., Bacon P., Bombardieri S., Hanly J., Hay E. (1996). The development and initial validation of the Systemic Lupus International Collaborating Clinics/American College of Rheumatology damage index for systemic lupus erythematosus. Arthritis Rheum..

[28] World Health Organization (2000). Obesity: Preventing and Managing the Global Epidemic: Report of a WHO Consultation.

[29] (2001). Expert Panel on Detection, Evaluation, and Treatment of High Blood Cholesterol in Adults Executive Summary of the Third Report of the National Cholesterol Education Program (NCEP) Expert Panel on Detection, Evaluation, and Treatment of High Blood Cholesterol in Adults (Adult Treatment Panel III). JAMA J. Am. Med. Assoc..

[30] Alberti K.G., Zimmet P.Z. (1998). Definition, diagnosis and classification of diabetes mellitus and its complications. Part 1: Diagnosis and classification of diabetes mellitus provisional report of a WHO consultation. Diabet. Med. J. Br. Diabet. Assoc..

[31] (1999). 1999 World Health Organization-International Society of Hypertension Guidelines for the Management of Hypertension. Clin. Exp. Hypertens..

[32] Romero-Martínez M., Shamah-Levy T., Cuevas-Nasu L., Méndez Gómez-Humarán I., Gaona-Pineda E.B., Gómez-Acosta L.M., Rivera-Dommarco J.Á., Hernández-Ávila M. (2017). Diseño metodológico de la Encuesta Nacional de Salud y Nutrición de Medio Camino 2016. Salud Pública México.

[33] Bernal-Orozco M.F., Vizmanos-Lamotte B., Rodríguez-Rocha N.P., Macedo-Ojeda G., Orozco-Valerio M., Rovillé-Sausse F., León-Estrada S., Márquez-Sandoval F., Fernández-Ballart J.D. (2013). Validation of a Mexican food photograph album as a tool to visually estimate food amounts in adolescents. Br. J. Nutr..

[34] Bourges R. H., Casanueva E., Rosado J.L. (2008). Recomendaciones de ingestión de nutrimentos para la población mexicana.

[35] Fernández-Gaxiola A.C., Bonvecchio Arenas A., Plazas Belausteguigoitia M., Kaufer-Horwitz M., Pérez-Lizaur A.B., Rivera Dommarco J. (2015). Guías Alimentarias y de Actividad física: En Contexto de Sobrepeso y Obesidad en la Población Mexicana: Documento de Postura.

[36] Institute of Medicine (2004). Dietary Reference Intakes for Water, Potassium, Sodium, Chloride, and Sulfate.

[37] Institute of Medicine (1998). Dietary Reference Intakes for Thiamin, Riboflavin, Niacin, Vitamin B_6_, Folate, Vitamin B_12_, Pantothenic Acid, Biotin, and Choline.

[38] FAO (2003). Diet, Nutrition, and the Prevention of Chronic Diseases: Report of a WHO-FAO Expert Consultation.

[39] Inano M., Pringle D.J. (1975). Dietary survey of low-income, rural families in Iowa and North Carolina. II. Family distribution of dietary adequacy. J. Am. Diet. Assoc..

[40] Sullivan K.M., Dean A., Soe M.M. (2009). On Academics: OpenEpi: A Web-Based Epidemiologic and Statistical Calculator for Public Health. Public Health Rep..

[41] Chaiamnuay S., Bertoli A.M., Fernández M., Apte M., Vil L.M., Reveille J.D., Alarcón G.S. (2007). The Impact of Increased Body Mass Index on Systemic Lupus Erythematosus: Data from LUMINA, a Multiethnic Cohort. JCR J. Clin. Rheumatol..

[42] Handono K., Sidarta Y.O., Pradana B.A., Nugroho R.A., Hartono I.A., Kalim H., Endharti A.T. (2014). Vitamin D prevents endothelial damage induced by increased neutrophil extracellular traps formation in patients with systemic lupus erythematosus. Acta Med. Indones..

[43] Pocovi-Gerardino G., Correa-Rodríguez M., Callejas-Rubio J.L., Ríos-Fernández R., Ortego-Centeno N., Rueda-Medina B. (2018). Dietary intake and nutritional status in patients with systemic lupus erythematosus. Endocrinol. Diabetes Nutr..

[44] Oeser A., Chung C.P., Asanuma Y., Avalos I., Stein C.M. (2005). Obesity is an independent contributor to functional capacity and inflammation in systemic lupus erythematosus. Arthritis Rheum..

[45] Fischer K., Przepiera-Będzak H., Sawicki M., Walecka A., Brzosko I., Brzosko M. (2017). Serum Interleukin-23 in Polish Patients with Systemic Lupus Erythematosus: Association with Lupus Nephritis, Obesity, and Peripheral Vascular Disease. Mediat. Inflamm..

[46] Sinicato N.A., Postal M., Peres F.A., Peliçari K.D.O., Marini R., dos Santos A.D.O., Ramos C.D., Appenzeller S. (2014). Obesity and Cytokines in Childhood-Onset Systemic Lupus Erythematosus. J. Immunol. Res..

[47] La Cava A. (2019). The Influence of Diet and Obesity on Gene Expression in SLE. Genes.

[48] Mobini M., Niksolat F., Mohammadpour R.A., Dashti Dargahloo S., Marzban D. (2018). Metabolic syndrome in patients with systemic lupus erythematosus: Association with disease activity, disease damage and age. Int. J. Rheum. Dis..

[49] Fujita Y., Fujii T., Mimori T., Sato T., Nakamura T., Iwao H., Nakajima A., Miki M., Sakai T., Kawanami T. (2014). Deficient Leptin Signaling Ameliorates Systemic Lupus Erythematosus Lesions in MRL/Mp- Fas lpr Mice. J. Immunol..

[50] Liu Y., Yu Y., Matarese G., La Cava A. (2012). Cutting Edge: Fasting-Induced Hypoleptinemia Expands Functional Regulatory T Cells in Systemic Lupus Erythematosus. J. Immunol..

[51] Versini M., Tiosano S., Comaneshter D., Shoenfeld Y., Cohen A.D., Amital H. (2017). Smoking and obesity in systemic lupus erythematosus: A cross-sectional study. Eur. J. Clin. Investig..

[52] Urowitz M.B., Gladman D., Ibañez D., Fortin P., Sanchez-Guerrero J., Bae S., Clarke A., Bernatsky S., Gordon C., Hanly J. (2007). Clinical manifestations and coronary artery disease risk factors at diagnosis of systemic lupus erythematosus: Data from an international inception cohort. Lupus.

[53] Urowitz M.B., Gladman D., Ibañez D., Fortin P., Sanchez-Guerrero J., Bae S., Clarke A., Bernatsky S., Gordon C., Hanly J. (2008). Accumulation of coronary artery disease risk factors over three years: Data from an international inception cohort. Arthritis Rheum..

[54] Symmons D.P.M., Gabriel S.E. (2011). Epidemiology of CVD in rheumatic disease, with a focus on RA and SLE. Nat. Rev. Rheumatol..

[55] Muthukumar A., Zaman K., Lawrence R., Barnes J.L., Fernandes G. (2003). Food restriction and fish oil suppress atherogenic risk factors in lupus-prone (NZB x NZW) F1 mice. J. Clin. Immunol..

[56] Hsieh C.-C., Lin B.-F. (2011). Dietary factors regulate cytokines in murine models of systemic lupus erythematosus. Autoimmun. Rev..

[57] Muller S., Quast T., SchrÃ¶der A., Hucke S., Klotz L., Jantsch J., Gerzer R., Hemmersbach R., Kolanus W. (2013). Correction: Salt-Dependent Chemotaxis of Macrophages. PLoS ONE.

[58] Duffy E.M., Meenagh G.K., McMillan S.A., Strain J.J., Hannigan B.M., Bell A.L. (2004). The clinical effect of dietary supplementation with omega-3 fish oils and/or copper in systemic lupus erythematosus. J. Rheumatol..

[59] Calder P.C. (2012). Long-chain fatty acids and inflammation. Proc. Nutr. Soc..

[60] Dankers W., Colin E.M., van Hamburg J.P., Lubberts E. (2017). Vitamin D in Autoimmunity: Molecular Mechanisms and Therapeutic Potential. Front. Immunol..

[61] Andreoli L., Dall’Ara F., Piantoni S., Zanola A., Piva N., Cutolo M., Tincani A. (2015). A 24-month prospective study on the efficacy and safety of two different monthly regimens of vitamin D supplementation in pre-menopausal women with systemic lupus erythematosus. Lupus.

[62] Kinoshita K., Kishimoto K., Shimazu H., Nozaki Y., Sugiyama M., Ikoma S., Funauchi M. (2010). Successful Treatment with Retinoids in Patients with Lupus Nephritis. Am. J. Kidney Dis..

[63] Vitales-Noyola M., Layseca-Espinosa E., Baranda L., Abud-Mendoza C., Niño-Moreno P., Monsiváis-Urenda A., Rosenstein Y., González-Amaro R. (2018). Analysis of Sodium Chloride Intake and Treg/Th17 Lymphocytes in Healthy Individuals and Patients with Rheumatoid Arthritis or Systemic Lupus Erythematosus. J. Immunol. Res..

[64] Wu C., Yosef N., Thalhamer T., Zhu C., Xiao S., Kishi Y., Regev A., Kuchroo V.K. (2013). Induction of pathogenic TH17 cells by inducible salt-sensing kinase SGK1. Nature.

